# Cryptic Diversity and Climatic Niche Divergence of *Brillia* Kieffer (Diptera: Chironomidae): Insights from a Global DNA Barcode Dataset

**DOI:** 10.3390/insects16070675

**Published:** 2025-06-27

**Authors:** Hai-Feng Xu, Meng-Yu Lv, Yu Zhao, Zhi-Chao Zhang, Zheng Liu, Xiao-Long Lin

**Affiliations:** 1 Engineering Research Center of Environmental DNA and Ecological Water Health Assessment, Shanghai Ocean University, Shanghai 201306, China; haifengxu2024@gmail.com (H.-F.X.); 2313307@st.shou.edu.cn (Y.Z.); zzc514644@gmail.com (Z.-C.Z.); 2 Shanghai Universities Key Laboratory of Marine Animal Taxonomy and Evolution, Shanghai Ocean University, Shanghai 201306, China; 3 Ecological and Environmental Monitoring Center of Xiong’an New Area, Xiong’an New Area 071800, China; mengyulvl@163.com; 4 Laboratory of Geo-Specimens Study and Testing, Geological Museum of China, Beijing 100034, China; 5 Department of Stratigraphy and Paleontology, Geological Museum of China, Beijing 100034, China

**Keywords:** cryptic species, DNA barcoding, COI

## Abstract

Accurate species identification of small aquatic insects remains challenging due to their morphological similarities. This study addresses this issue by developing a DNA barcode reference library for the globally distributed *Brillia* (Diptera: Chironomidae). We analyzed cytochrome *c* oxidase subunit I (COI) sequences of 241 specimens belonging to 13 *Brillia* species from 18 countries, including 56 newly generated and 185 publicly available COI barcodes. Our integrated approach included genetic distance analysis, haplotype network construction, and ecological niche modeling. The results revealed remarkable cryptic diversity, with sequences clustering into 30 Barcode Index Numbers and 158 unique haplotypes, most being region-specific. Notably, East Asian and North American populations showed complete genetic distinctness, suggesting long-term isolation. Environmental factors, particularly temperature and precipitation gradients, were identified as key drivers of this diversification. The study also corrected several misidentifications in existing databases. These findings significantly advance our understanding of *Brillia* diversity and provide a reliable molecular tool for freshwater ecosystem monitoring, with important implications for biodiversity conservation and environmental assessment.

## Simple Summary

Identifying small and ecologically important insects has always been challenging. Our study focused on the globally distributed *Brillia*, using DNA technology to develop improved identification methods. By analyzing 241 COI barcodes from 18 countries, we discovered these seemingly similar midges actually harbor remarkable hidden diversity, revealing 158 distinct genetic types. Interestingly, *Brillia* from East Asia and North America showed substantial genetic differences, likely resulting from long-term adaptation to different environments. Temperature and precipitation were identified as key factors driving these variations. These findings not only help scientists more accurately identify midge species, but also enable better monitoring of freshwater ecosystem health, providing significant value for water resource and biodiversity conservation efforts.

## 1. Introduction

Global biodiversity is undergoing an unprecedented decline due to climate change and human activities [1,2], threatening ecosystem stability and sustainability [3]. Effective conservation strategies require accurate species identification and distribution data [4,5], yet many species remain undescribed, especially in biodiversity hotspots [6]. Freshwater ecosystems, among the most threatened globally, demand precise species monitoring [7]. Traditional morphology-based methods maybe inefficient and inconsistent [4], while DNA barcoding offers a robust alternative. This technology uses standardized genetic markers for species identification [8,9], excelling at detecting cryptic diversity [10,11,12]. Integrated with environmental DNA (eDNA) analysis, it enables efficient, sensitive, and cost-effective aquatic monitoring [13], including multi-taxa detection from single samples [14,15], aiding conservation policies [4]. However, DNA barcoding and eDNA metabarcoding rely on comprehensive reference databases. While Europe has advanced freshwater invertebrate barcode libraries [16,17,18], Asia—with a higher diversity and taxonomic complexity—lags behind, particularly for groups like caddisflies, lacewings, mayflies, and chironomids [19,20,21,22,23,24,25,26,27,28,29]. Expanding regional barcode databases is thus critical for improving biodiversity monitoring.

Against this backdrop, Chironomidae (Diptera) have emerged as a focal group for biodiversity assessment and ecological monitoring, being one of the most diverse benthic invertebrate taxa globally. Currently, over 7800 described species are documented [30,31,32], distributed across all zoogeographical regions, including extreme ecosystems such as Antarctica [33,34]. Chironomids not only play crucial ecological roles in freshwater ecosystems but also serve as sensitive bioindicators of environmental change [31]. Their exceptional diversity and broad environmental adaptability make them ideal models for studying phylogenetic relationships and biogeographic patterns [35,36,37,38]. However, traditional morphology-based classification of chironomids faces significant challenges, including ambiguous interspecific boundaries, laborious identification procedures, and strong subjectivity, leading to a substantial underestimation of their true diversity [39,40]. Current estimates suggest the actual number of chironomid species may exceed 20,000 [41], far surpassing currently described records. The application of DNA barcoding technology in this group has not only dramatically improved species identification efficiency but also provided powerful tools for phylogenetic studies and ecological function analysis. Nevertheless, rather than replacing morphology-based taxonomy, DNA barcoding is most effective when used in conjunction with traditional approaches. Integrative taxonomy, which combines morphological, molecular, and ecological data, is increasingly recognized as the most robust framework for delineating species and understanding their evolutionary relationships [42,43].

**Figure 1:**
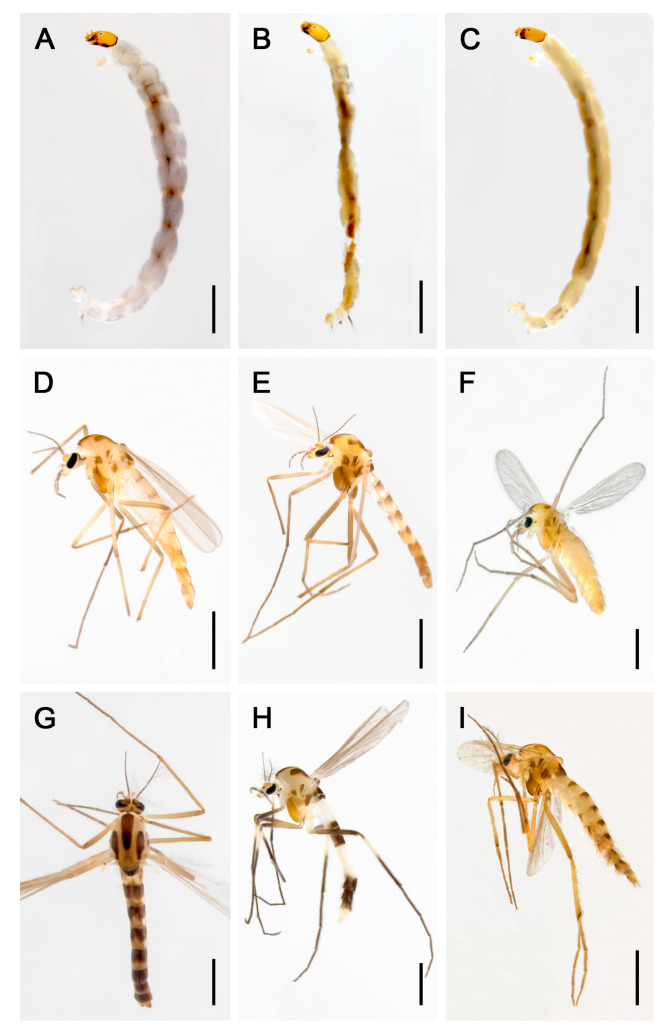
Photos of *Brillia*: (**A**) larva of *Brillia* sp. 2XL; (**B**) larva of *Brillia japonica* Tokunaga, 1939; (**C**) larva of *Brillia bifasciata* Wang, Zheng & Ji, 1994; (**D**) adult male of *Brillia* sp. 1XL; (**E**) adult male of *Brillia japonica*; (**F**) adult female of *Brillia japonica*; (**G**) adult male of *Brillia longifurca* Kieffer, 1921; (**H**) adult male of *Brillia bifasciata*; (**I**) adult male of *Brillia brevicornis* Wang, Zheng & Ji, 1994. Scale bars = 1 mm. Photos were photographed by Xiao-Long Lin, Shanghai Ocean University.

*Brillia* Kieffer, 1913 (Figure 1), a representative genus within Orthocladiinae, was established with the species *Metriocnemus bifidus* Kieffer, 1909, and currently includes 21 extant species in Holarctic and Oriental regions and one extinct species from Eocene Rovno amber in Ukraine, dated to approximately 33.9–40 million years ago [44,45,46,47,48]. *Brillia* are divided into two species groups (the *flavifrons* group and the *modesta* group) and are characterized in adults by bare eyes, antennal ratio (AR) typically <1.5, R_4+5_ wing vein branching near the apex, and a male tergite IX with a denticulate or pectinate posterior margin and reduced superior volsella. Larvae feature well-developed and often pigmented thoracic horns, a third antennal segment shorter than one-third of the total antenna length, mandibles with 3–4 blunt teeth, banded ventro-lateral setae on the abdomen, short digitiform or globular anal gills, and generally dark brown body coloration [44,45,46,49]. Diagnostic traits such as undeveloped superior volsella and wing vein branching pattern help distinguish *Brillia* from related genera such as *Xylotopus* Oliver, 1982, and *Euryhapsis* Oliver, 1981 [50,51].

*Brillia* species inhabit depositional zones of rivers, streams, and small lentic waters, in which larvae feed primarily on humic substances, contributing to material cycling in freshwater ecosystems [46]. Despite there being a morphological foundation for taxonomy, global systematic research remains limited, partly due to insufficient species surveys in the Oriental and Afrotropical regions [52] and the challenge of linking adult and larval stages [44]. Taxonomy traditionally emphasizes male adult traits such as AR, body color, and genitalia, but these often exhibit considerable intraspecific variation and are less informative for females and larvae. Most recent descriptions still focus on male morphology, constraining accurate identification. Although molecular techniques offer promising tools for phylogenetic and species delimitation studies, current molecular data remain sparse and inadequate for resolving the evolutionary framework of the genus [53].

To address these gaps, this study aims to construct a global DNA barcode reference database for *Brillia* with three main objectives: (1) providing molecular evidence for matching different life stages; (2) revealing cryptic species diversity; and (3) correcting misidentifications in current classifications. In addition to these overarching goals, this paper specifically focuses on identifying cryptic diversity within *Brillia* and investigating ecological niche divergence across broad geographic regions. The resulting database will compensate for the limitations of traditional morphological taxonomy, provide reliable molecular tools for freshwater ecological monitoring and biodiversity assessment, and lay the groundwork for future research on ecological functions.

## 2. Materials and Methods

Between 2014 and 2020, 56 specimens belonging to nine *Brillia* species were collected from the field in China and Italy using Malaise traps, light traps, and D-nets. Adult and larval specimens were preserved in 85% and 95% ethanol, respectively, and stored in the dark at 4 °C. Morphological identification was conducted under a stereomicroscope, with some specimens dissected and examined using a compound microscope. Taxonomic assignments were made based on relevant literature on *Brillia* [44,46,49,54]. All vouchers are currently deposited in the College of Fisheries and Life Science, Shanghai Ocean University.

For each adult specimen, muscle tissue was extracted from legs or the thorax for DNA extraction, while for larvae, abdominal and thoracic muscle tissues were used after gut removal. DNA was extracted using the Qiagen DNA Blood and Tissue Kit (Qiagen, Hilden, Germany) and Universal Genomic DNA Kit (CWBIO, Taizhou, China), following the manufacturer’s protocol. The COI barcode region was amplified using the universal primers LCO1490 and HCO2198 [55], and PCR conditions followed a previous study [28]. PCR products were purified and subjected to Sanger sequencing. All specimens were ultimately slide-mounted in Euparal following standard procedures [45], with key morphological structures preserved for taxonomic identification, morphometric analysis, and morphological description.

In addition, 185 public COI sequences of *Brillia* were retrieved from the Barcode of Life Data System (BOLD) [56]. Sequences were filtered to retain only those ≥500 bp in length, excluding those containing stop codons, flagged with BOLD quality warnings, or lacking a valid Barcode Index Numbers (BINs) assignment (data accessed on 13 April 2025). A total of 185 public sequences and 56 newly generated sequences were compiled, forming a dataset of 241 sequences, titled “Global *Brillia* COI DNA barcodes (DS-BRICOI)”, which was deposited in BOLD. Raw sequencing data were assembled and quality-checked using Geneious Prime v2022.2.2 (Biomatters, Auckland, New Zealand). Multiple sequence alignment was performed in MEGA X (Philadelphia, PA, USA) [57] using the MUSCLE algorithm (Edmonds, WA, USA) [58], with stop codons and translation errors carefully screened. The p-distance method [59] was employed to calculate genetic distances of COI gene sequences using the BioPython module (Cambridge, UK) [60] in Python v3.11 (Wilmington, DE, USA) [61].

A neighbor-joining phylogenetic tree was constructed under the Kimura 2-parameter (K2P) model (Kyoto, Japan) [62], with 1000 bootstrap replicates and the pairwise deletion method to assess branch support. Additionally, species delimitation was conducted using the ABGD method (Marseille, France) [63] under the K2P model with a minimum prior intraspecific divergence (P_min_) set to 0.005 (analysis date: 11 May 2025). To investigate potential cryptic differentiation, a COI haplotype network was constructed using the TCS method (Ithaca, NY, USA) [64] implemented in PopART v1.7 (Dunedin, New Zealand) [65], with missing data and insertion sites excluded from the analysis.

To investigate the potential ecological niche differentiation and geographic distribution patterns of *Brillia* species, a range of environmental variables were collected, encompassing key dimensions such as climate, moisture, and topography. The data included 19 bioclimatic variables [66], frost frequency and duration [66], aridity index [67], annual mean cloud cover [68], and topographic variables including elevation and slope [69]. All environmental variables were obtained in GeoTIFF (.tif) or NetCDF (.nc) formats from databases such as CHELSA, CGIAR-CSI, and EarthEnv. Environmental variables were standardized in R v4.3.2 (Vienna, Austria) [70], followed by principal component analysis (PCA) to explore the distribution patterns of samples along environmental gradients. Based on the first two PCA axes, a clustering plot of sample points was generated, and ecological niche distributions of different geographic groups were visualized in temperature-precipitation space using kernel density estimation (KDE). Samples were divided into four geographic regions: East Asia (EA), Northern Europe (NE), Central Europe (CE), and North America (NA). In combination with haplotype network structures, the responses of different groups to key environmental variables were compared.

## 3. Results

### 3.1. Dataset Characteristics and Global Distribution

**Figure 2:**
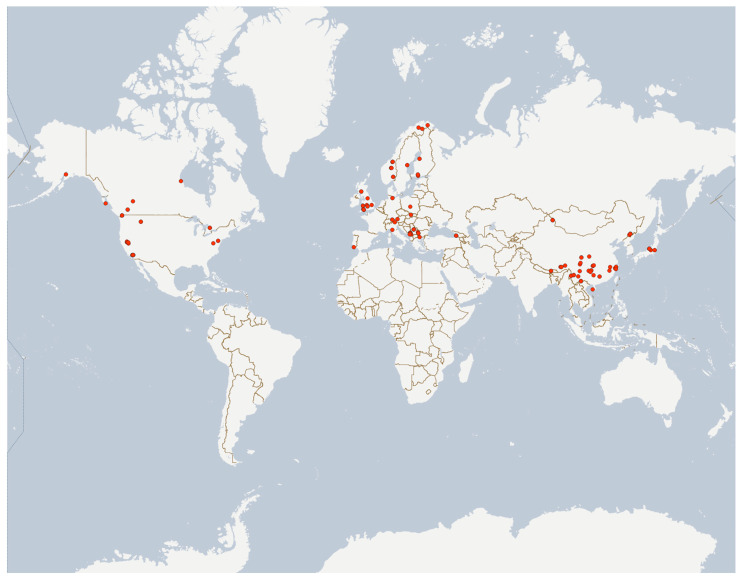
Geographical distribution of *Brillia* specimens in this study.

All 241 COI barcode sequences ranged in length from 507 to 658 bp, including 65 complete 658 bp barcode sequences. The samples covered a broad altitudinal range from three to 3290 m and spanned a wide geographical distribution between 19.083° N to 70.426° N latitude and 151.081° W to 140.165° E longitude (Figure 2). These sequences were classified into 30 BINs, comprising 20 concordant BINs, 9 singleton BINs, and 1 discordant BIN.

The samples showed a markedly uneven distribution across four major regions. CE accounted for the largest proportion (89 sequences, 36.9%), primarily collected from Balkan Peninsula regions including Batumi, Georgia (20 sequences), Montenegro (9 sequences), and Serbia (8 sequences). NA represented the second largest group (81 sequences, 33.6%), with concentrations along the western coastal areas of the United States and Canada. In contrast, EA and NE had significantly fewer samples, constituting only 15.8% (38 sequences) and 13.7% (33 sequences), respectively. The EA samples were mainly from southwestern China (Yunnan, Xizang, etc.), while NE specimens were predominantly collected from subarctic regions such as northern Norway.

### 3.2. Species Clustering

**Figure 3:**
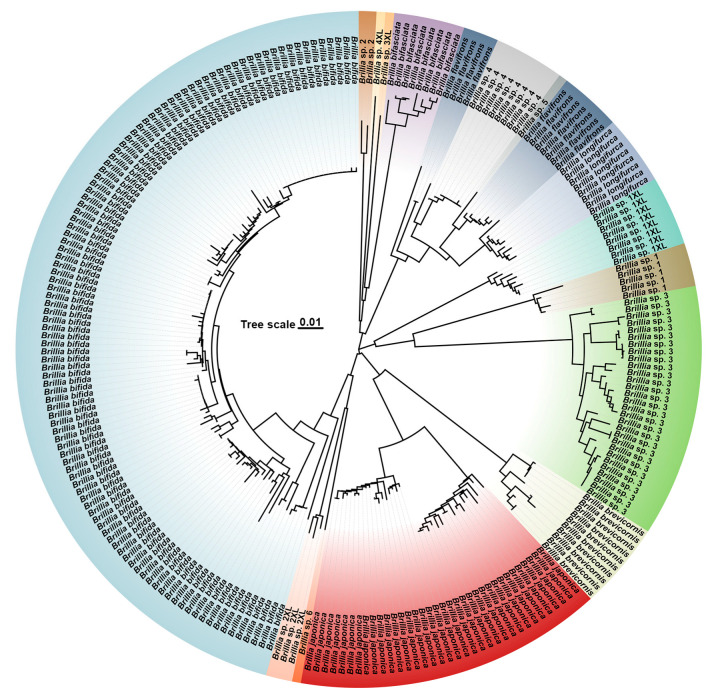
Neighbor-joining tree constructed based on the COI barcodes of *Brillia*.

The neighbor-joining (NJ) phylogenetic tree constructed from COI sequences (Figure 3) recovered 13 putative *Brillia* species. Genetic distance analysis revealed a maximum intraspecific distance of 0.093 in *Brillia bifida* Kieffer, 1909 [52], and a minimum interspecific distance of 0.095 between *Brillia* sp. 2XL and *Brillia* sp. 6. The phylogenetic results demonstrated that most taxa formed well-supported monophyletic clades corresponding to morphological classification units.

*Brillia bifida* represented the most abundant samples, forming a large monophyletic cluster with clear intraspecific consistency. *B.* sp. 2XL and *B.* sp. 6 formed two distinct but closely related sister clades. *Brillia japonica*, Tokunaga, 1939 [52], and *Brillia brevicornis* Wang, Zheng & Ji, 1994 [46], formed adjacent clades within a major branch, indicating shared genetic background. *Brillia* sp. 3, *Brillia* sp. 1 and *Brillia* sp. 1XL collectively formed a distinct clade, while *B.* sp. 3 itself exhibited multiple subclades with internal genetic structure, suggesting potential intraspecific differentiation. *Brillia* sp. 3XL and *Brillia* sp. 4XL formed a distinct clade, respectively. *Brillia bifasciata* Wang, Zheng & Ji, 1994 [46], and *Brillia* sp. 2 each constituted separate monophyletic branches. Notably, *Brillia* sp. 4 and *Brillia* sp. 5 were embedded within *Brillia flavifrons,* Johannsen, 1905, while one lineage of *B. flavifrons* showed paraphyly with *Brillia longifurca,* Kieffer, 1921 [52].

### 3.3. Species Delimitation and Genetic Divergence Revealed by ABGD Analysis

**Figure 4:**
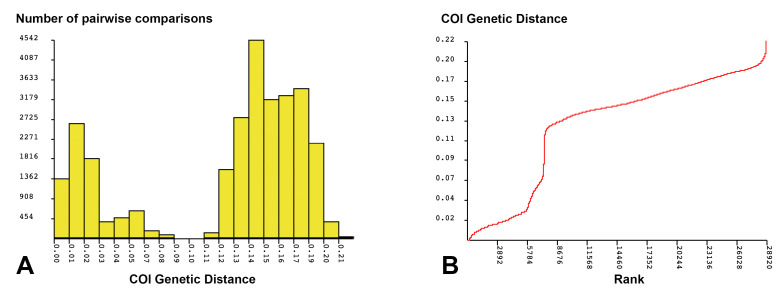
ABGD analysis of COI sequence for *Brillia*. (**A**) Distribution of pairwise genetic distances based on the number of comparisons; (**B**) rank-ordered curve of pairwise genetic distances.

Under the maximum intraspecific divergence threshold (*p* = 0.022), the ABGD analysis identified 13 molecular operational taxonomic units (mOTUs, Table S1). The results revealed a distinct barcode gap (barcode gap distance = 0.095), providing strong evidence for species-level genetic divergence (Figure 4). Notably, *B.* sp. 6, *B.* sp. 3XL, and *B* sp. 4XL were each assigned to separate mOTUs, indicating clear genetic differentiation.

The figure showed a marked distinction between inter and intraspecific K2P genetic distances. The number of pairwise differences dropped to zero at the 9% threshold, rose again between 11% and 12%, and continued to increase, peaking around 14% and 15%. All pairwise comparisons were grouped at the species level below the 5% threshold, and within-genus relationships were retained between 6% and 9%. When intraspecific divergence exceeds 10%, cryptic species may exist despite morphological similarity [19,71]. A distinct inflection point around 0.12 in the COI genetic distance plot further supports the discontinuity between intra- and interspecific variation. These findings demonstrate that ABGD effectively delineates potential species units based on genetic divergence.

As the prior intraspecific divergence threshold increased, the number of mOTUs gradually converged and stabilized at 13 units, indicating the presence of cryptic species within the dataset. Among the identified units, the mOTU corresponding to *B. bifida* contained the largest number of sequences (n = 110). The remaining sequences were grouped into nine different mOTUs, including 29 sequences of *B.* sp. 3, 35 of *B. japonica*, 8 of *B. bifasciata*, 3 of *B.* sp. 2XL, 10 of *B. brevicornis*, 8 of *B.* sp. 1XL, 5 of *Brillia* sp. 1, and 2 of *B.* sp. 2. Additionally, *B.* sp. 4, *B.* sp. 5, *B. flavifrons*, and *B. longifurca* were grouped into a single mOTU, with a total of 28 sequences.

### 3.4. Haplotype Diversity and Geographic Specificity

A total of 158 unique haplotypes were identified among the analyzed *Brillia* samples (Figure S1), representing specimens from 18 countries across Europe and North America. The haplotypes displayed high diversity, with most being singletons and only a few shared among individuals or populations. Haplotype network analysis revealed significant differences in haplotype frequency—some were represented by up to 10 samples, while most were unique to individual specimens. Notably, even geographically close populations (e.g., those from Central Europe) exhibited distinct haplotypes.

The results showed a strong geographic specificity in haplotype distribution. Regional groups generally possessed region-specific haplotypes; for instance, no haplotypes were shared between EA and NA, and only limited peripheral connections were observed between NE and CE.

### 3.5. Niche Differentiation of Brillia Geographic Groups

PCA based on 27 standardized environmental variables, including BioClim climate variables, frost characteristics, aridity index, mean annual cloud cover, and topographic variables, revealed significant differentiation of *Brillia* samples along environmental gradients. The environmental variables with the highest loadings on PC1 and PC2 included mean annual temperature (bio1), minimum temperature in the coldest month (bio6), mean temperature in the coldest quarter (bio11), annual precipitation (bio12), precipitation in the driest quarter (bio17), as well as the aridity index and cloud cover. The first two principal components, PC1 (temperature gradient) and PC2 (moisture gradient), explained 39.1% and 17.6% of the environmental variation, respectively, accounting for a total of 56.7% of the overall variation. These variables primarily represent a combined gradient of temperature and moisture.

**Figure 5:**
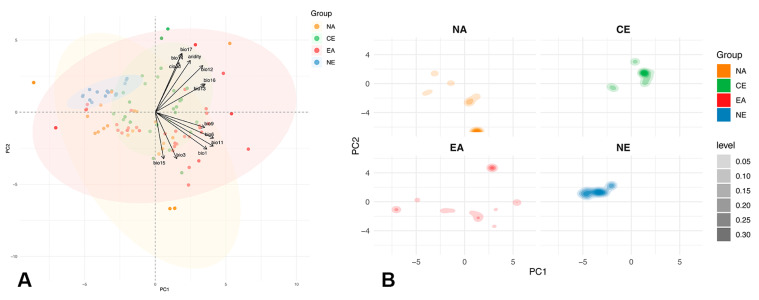
Ecological niche distribution patterns of *Brillia* species along environmental gradients. (**A**) PCA plot based on standardized environmental variables (EA: East Asia, NE: Northern Europe, CE: Central Europe, NA: North America); (**B**) KDE plot of different geographic groups in the two-dimensional space of temperature and precipitation.

The PCA biplot showed that populations from CE and EA exhibited a wide distribution along PC1, indicating a broad range of temperature–precipitation variation; in contrast, the NA group was mainly concentrated in the negative quadrant of PC2, and the NE group clustered in the negative region of PC1, indicating relatively narrower niche spaces. Further kernel density estimation (KDE) analyses confirmed niche differentiation among groups. In the temperature-precipitation two-dimensional space, ecological niches of different geographic groups showed minimal overlap: EA and CE were distributed in a moderate-humid intermediate zone, NE favored cold and wet areas, while NA concentrated in cold and dry regions (Figure 5). These results support a clear ecological separation of populations across the four regions.

## 4. Discussion

### 4.1. Geographic Distribution of Brillia

This study conducted a systematic analysis of the geographic distribution patterns of *Brillia* and revealed that the genus exhibits a distinct intercontinental distribution, primarily spanning major regions of the Northern Hemisphere, with only one verified record (Peninsular Malaysia) marginally extending into the Southern Hemisphere based on Global Biodiversity Information Facility (GBIF, https://www.gbif.org) occurrence data (data accessed on 13 April 2025) [52]. In terms of spatial distribution, *Brillia* demonstrates strong environmental adaptability. Along the latitudinal gradient, its distribution ranges from the mid to high latitude regions of NE and NA to the subtropical–temperate transitional zones of EA. Along the environmental gradient, it occupies diverse habitats, including the cold climate zones of NE, the temperate and humid areas of CE and NA, and the topographically complex regions of EA. This broad distribution pattern is highly consistent with the climatic niche differentiation revealed by principal component analysis. Populations in NE and EA are mainly associated with environments characterized by high frost frequency and harsh winters, while those in CE and NA are more adapted to warmer, humid habitats with higher mean annual cloud cover. The spatial heterogeneity of environmental factors—such as temperature, precipitation, frost frequency, and aridity—may jointly drive the ecological niche differentiation observed among regional populations of *Brillia* [72,73].

Notably, the EA population inhabits topographically complex areas such as the Hengduan Mountains. These regions may offer unique microrefugia, potentially serving as an ecological basis for the genus’s ability to span different climatic zones [73]. However, the current lack of samples from the Southern Hemisphere limits our understanding of the genus’ global distribution, underscoring the need for future investigations into its potential southern range.

### 4.2. Taxonomic Challenges Revealed by ABGD and NJ Analysis

ABGD analysis revealed complex taxonomic issues within Group 7. Specifically, *B.* sp. 5 (GMFRN026-15) formed a distinct BIN but was assigned to the same mOTU as *B. flavifrons*. This pattern may indicate a cryptic species that is morphologically similar to *B*. *flavifrons* but is genetically divergent [74]. Notably, *B*. *flavifrons* exhibits a pronounced geographic specificity in its distribution, being mainly confined to the Nearctic and eastern Palearctic regions—particularly Japan and the Russian Far East [75]. Historical records from Europe may largely stem from misidentifications involving *B. longifurca* [52,76]. These findings collectively suggest that *B*. *flavifrons* may have a more complex pattern of geographic population structure than previously recognized, and that its intraspecific genetic diversity has likely been underestimated in earlier studies. To resolve the taxonomy of these closely related lineages, more comprehensive studies are required, including broader geographic sampling, multilocus analyses, and detailed morphological comparisons [77]. Further analysis showed that both *B.* sp. 4 and *B.* sp. 5 were deeply embedded within the *B. flavifrons* clade. Given their collection localities, these specimens likely represent *B. flavifrons* individuals. However, the absence of corresponding voucher specimens precludes definitive morphological confirmation. Additionally, minimal genetic differentiation between *B. longifurca* and *B. flavifrons* suggests these may represent synonymous taxa.

In conjunction with the NJ tree results, this study also corrected several misidentifications in public databases. Eight samples previously identified as *Euryhapsis* sp. were reclassified as *B.* sp. 4, while three samples originally assigned only to Chironomidae were revised to *B. bifida* (SIIP6737-24 and SIIP7145-24) and *B.* sp. 2 (BIOAI046-14), respectively (Table S1). These results underscore the inconsistencies between BIN and mOTU classification standards and highlight the limitations of current reference databases and molecular markers [78,79]. Accurate taxonomic resolution of these groups will require integrative approaches that combine morphological traits, multilocus data, and broader sample verification, while also carefully considering the effects of marker choice and clustering methods on species delimitation outcomes.

### 4.3. Geographic Differentiation and Localized Haplotypes in Brillia

Among the 158 haplotypes identified in this study, the vast majority were singletons, with only a few shared among individuals, indicating the extremely high genetic diversity within *Brillia* [80]. The dominance of singleton haplotypes suggests pronounced genetic differentiation among geographically isolated populations, possibly due to limited dispersal ability [81].

Species of *Brillia* are typically associated with cold-water and stream habitats, and such habitat specialization may restrict their dispersal and promote population divergence [44,46]. Several haplotypes were geographically restricted, such as Hap_76 (Bulgaria), Hap_25 (Georgia), Hap_41 (Japan), and Hap_92 (China), suggesting possible local differentiation or even cryptic species, especially in mountainous or otherwise geographically isolated regions [82]. Notably, there was no clear clustering of haplotypes between continents, which may reflect postglacial recolonization, habitat fragmentation, or anthropogenic disturbances shaping their historical biogeographic patterns [83,84,85].

### 4.4. Ecological Niche Divergence Driven by Climatic and Topographic Factors

Results from environmental PCA and KDE jointly reveal marked ecological niche differentiation among *Brillia* populations across geographic regions. The primary environmental gradients—especially those related to temperature and moisture, such as minimum temperature of the coldest month, precipitation seasonality, and aridity index—play a key role in shaping population-level ecological distributions [86]. Local adaptations along these gradients may promote speciation and geographic isolation [87].

Populations from EA and CE exhibit a broad distribution in environmental space, suggesting their adaptation to a wide range of ecological conditions, from humid and temperate to relatively dry climates. In contrast, NE populations are concentrated in cold regions with high frost frequency, indicating a strong ecological preference for colder environments and an enhanced capacity for extreme climate tolerance. NA populations occupy a distinct ecological space, characterized by higher aridity and more pronounced precipitation seasonality, suggesting the evolution of unique ecological strategies under different climatic regimes [88].

This pattern of ecological differentiation aligns well with the geographic distribution of the samples, providing *Brillia* with diverse opportunities for environmental adaptation. It also likely contributes to geographic isolation and ecological divergence among populations, which may explain the strong genetic differentiation observed in the haplotype structure [89].

## 5. Conclusions

This study presents the first global DNA barcode reference library for *Brillia*, encompassing 241 COI sequences from 18 countries. The results reveal remarkable genetic diversity within the genus, including 158 haplotypes, the majority of which are singletons. The delineation of 30 BINs and 13 mOTUs indicates the presence of potential cryptic species. The haplotype structure shows clear geographic patterns, with almost no shared haplotypes between East Asian and North American populations, suggesting long-term isolation and limited dispersal capacity in these cold-stream-adapted lineages. Ecological niche analyses indicate that temperature and moisture gradients are key drivers of population divergence, with distinct adaptive differentiation observed among European, Asian, and North American lineages. The detection of high haplotype diversity in previously poorly surveyed regions, such as China, highlights critical knowledge gaps in current biodiversity assessments. By integrating molecular data with ecological variables, this study provides a valuable foundation for future taxonomic revisions and biomonitoring efforts. Moreover, it offers new insights into how freshwater insects adapt to large-scale geographic and environmental heterogeneity.

## Data Availability

A list of all species, specimens, their individual images, georeferences, primers, sequences, and other relevant laboratory data of all *Brillia* specimens are available through the public dataset “Global *Brillia* COI DNA barcodes (DS-BRICOI)” in the Barcode of Life Data System (http://www.boldsystems.org).

## Supplementary Materials

The following supporting information can be downloaded at: https://www.mdpi.com/article/10.3390/insects16070675/s1, Figure S1. Haplotype diversity of Brillia in this study; Table S1. The list of Brillia specimens incorporates revised taxonomic designations and mOTU assignments based on ABGD analysis.
