# Supplementary figures and images for: Cryptic Diversity and Climatic Niche Divergence of Brillia Kieffer (Diptera: Chironomidae): Insights from a Global DNA Barcode Dataset
Source: Insects. 2025 Jun 27;16(7):675. doi: 10.3390/insects16070675 (PMC12294968; doi:10.3390/insects16070675)

# COI haplotype network of *Brillia* all over the world

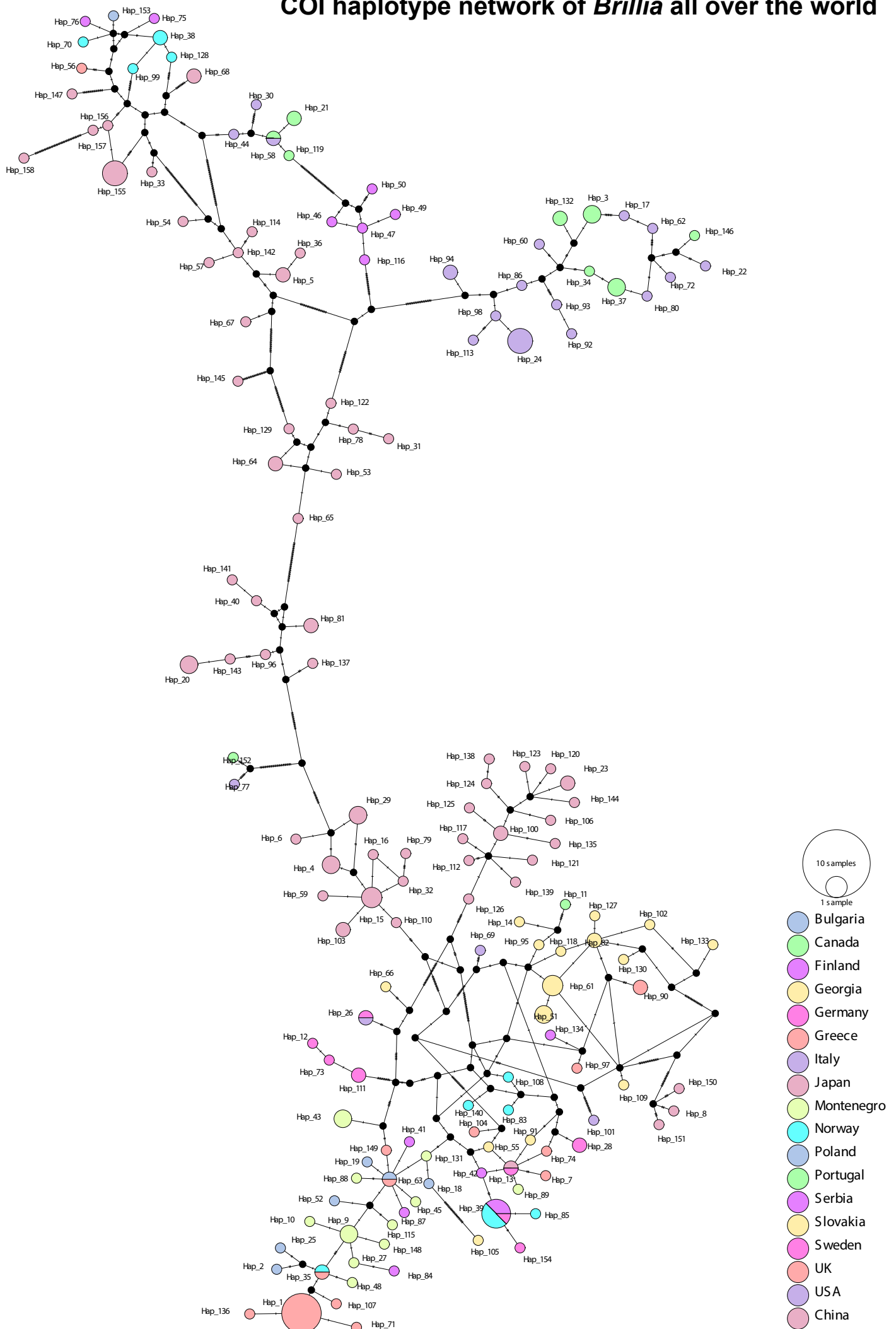

Supplement: Supplementary file 1 [file insects-16-00675-s001.zip › Figure S1.pdf]
